# Cloning and Characterization of Two Potent Kunitz Type Protease Inhibitors from *Echinococcus granulosus*

**DOI:** 10.1371/journal.pntd.0004268

**Published:** 2015-12-08

**Authors:** Shiwanthi L. Ranasinghe, Katja Fischer, Wenbao Zhang, Geoffrey N. Gobert, Donald P. McManus

**Affiliations:** 1 Molecular Parasitology Laboratory, QIMR Berghofer Medical Research Institute, Herston, Queensland, Australia; 2 School of Public Health, The University of Queensland, Herston, Queensland, Australia; 3 Department of Biochemistry, Xinjiang Medical University, Urumqi, Xinjiang, China; 4 Clinical Medical Research Institute, The First Affiliated Hospital of Xinjiang Medical University, Urumqi, Xinjiang, China; University of Cambridge, UNITED KINGDOM

## Abstract

The tapeworm *Echinococcus granulosus* is responsible for cystic echinococcosis (CE), a cosmopolitan disease which imposes a significant burden on the health and economy of affected communities. Little is known about the molecular mechanisms whereby *E*. *granulosus* is able to survive in the hostile mammalian host environment, avoiding attack by host enzymes and evading immune responses, but protease inhibitors released by the parasite are likely implicated. We identified two nucleotide sequences corresponding to secreted single domain Kunitz type protease inhibitors (EgKIs) in the *E*. *granulosus* genome, and their cDNAs were cloned, bacterially expressed and purified. EgKI-1 is highly expressed in the oncosphere (egg) stage and is a potent chymotrypsin and neutrophil elastase inhibitor that binds calcium and reduced neutrophil infiltration in a local inflammation model. EgKI-2 is highly expressed in adult worms and is a potent inhibitor of trypsin. As powerful inhibitors of mammalian intestinal proteases, the EgKIs may play a pivotal protective role in preventing proteolytic enzyme attack thereby ensuring survival of *E*. *granulosus* within its mammalian hosts. EgKI-1 may also be involved in the oncosphere in host immune evasion by inhibiting neutrophil elastase and cathepsin G once this stage is exposed to the mammalian blood system. In light of their key roles in protecting *E*. *granulosus* from host enzymatic attack, the EgKI proteins represent potential intervention targets to control CE. This is important as new public health measures against CE are required, given the inefficiencies of available drugs and the current difficulties in its treatment and control. In addition, being a small sized highly potent serine protease inhibitor, and an inhibitor of neutrophil chemotaxis, EgKI-1 may have clinical potential as a novel anti-inflammatory therapeutic.

## Author Summary

The hydatid tapeworm *Echinococcus granulosus* is able to survive in its mammalian hosts for many years without being digested by proteases. Two *E*. *granulosus* Kunitz proteins with potent protease inhibitory properties were identified and characterized. These Kunitz proteins may provide protection to the parasite from proteolytic digestion. These newly identified proteins are promising targets for developing new control interventions against echinococcosis, and one has potential as a novel anti-inflammatory therapeutic.

## Introduction

The dog tapeworm *Echinococcus granulosus* is one of a group of medically important parasitic helminths of the family Taeniidae (Platyhelminthes; Cestoda; Cyclophyllidea). Its life cycle involves two mammals: an intermediate host, usually a domestic or wild ungulate, with humans being accidental hosts, and a canine definitive host such as the domestic dog. The larval metacestode stage causes cystic echinococcosis (CE) (hydatidosis; cystic hydatid disease), a chronic cyst-forming disease in the intermediate/human host [1]. Hermaphrodite adult worms of *E*. *granulosus* reside in the small intestine of canines and pass eggs containing embryos (oncospheres) in feces. Following ingestion by a human or an intermediate host such as a sheep, the egg hatches in the intestine to release the oncosphere which penetrates through the gut wall and is carried in the blood system to various internal organs, mainly the liver or lungs, where it develops into a hydatid cyst. Dogs and other canines get infected by eating offal with fertile hydatid cysts containing larval protoscoleces. These larvae evaginate, attach to the gut, and develop into 3–6 mm long adult parasites which reach sexual maturity 4–5 weeks later [2]. The molecular mechanisms whereby the adult worms are able to survive in the dog gut without being damaged from host intestinal proteases and how oncospheres evade host immune attack in the blood system remain largely unknown. However, the recent deciphering of the *E*. *granulosus* genome and transcriptome [3, 4] provides insights as to how these processes might occur.

Kunitz type proteins, which belong to the I2 family of protease inhibitors, have been characterized from many organisms including sea anemone [5], cone snail [6], scorpion [7], spider [8], ticks and biting flies [9, 10], parasitic helminths [11–13] and mammals [14]. Bovine pancreatic trypsin inhibitor (BPTI) is the classic member of this family of proteins and was the first Kunitz-type protease inhibitor described [15]. In invertebrates, Kunitz inhibitors are involved in various physiological processes such as blood coagulation, fibrinolysis, inflammation and ion channel blocking with or without protease inhibition [16]. These proteins possess one or more Kunitz domains; the Kunitz-type motif consists of around 60 amino acids and has six conserved cysteine residues which connect with three disulphide bonds in a characteristic pattern (C1-C6, C2-C4, and C3-C5). The C1-C6 and C3-C5 bonds are required for the maintenance of native confirmation [17] whereas the C2-C4 bond stabilizes the folded structure [18]. Position P_1_ [19] of the reactive site is the major determinant of the energetic and specificity of protease recognition by Kunitz inhibitors [20].

A previous study described a multigene family of eight (EgKU1-EgKU8) secreted monodomain Kunitz proteins from *E*. *granulosus* protoscoleces preferentially expressed by pepsin/H (+)-treated worms [21]. Structural modeling revealed EgKU1 was a cation-channel blocker but only EgKU8 behaved as a potential protease inhibitor suggesting the majority of these Kunitz proteins were involved in functions other than protease inhibition [21].

By interrogation of the available genome sequence data for *E*. *granulosus* [3, 4] we identified two gene sequences (designated *EgKI-1* and *EgKI-2*) encoding two polypeptides similar to single domain Kunitz proteins. The two cDNAs were expressed in *Escherichia coli*, and the recombinant proteins (rEgKI-1 and rEgKI-2) were purified and functionally characterized. EgKI-2 reacted as a typical trypsin inhibitor, whereas EgKI-1, a potent inhibitor of chymotrypsin and neutrophil elastase, was able to significantly reduce neutrophil infiltration in the λ-carrageenan mouse air pouch model of local inflammation, and is the first Kunitz type serine protease inhibitor shown to bind calcium.

## Materials and Methods

### Identification and expression of EgKI-1 and EgKI-2

Two nucleotide sequences (*EgKI-1* and *EgKI-2*), encoding single domain Kunitz type protease inhibitors, were identified by interrogation of available *E*. *granulosus* genomic sequence for Kunitz domains. Searches for similar nucleotide sequences were performed using BLAST (http://blast.ncbi.nlm.nih.gov/Blast.cgi) on the NCBI (National Centre for Biotechnology Information) web site. The presence of a signal sequence in both proteins was checked using signalP 4.1 (http://www.cbs.dtu.dk/services/SignalP/) [22]. Protein domains were identified by searching the PROSITE database (http://prosite.expasy.org/) [23] and multiple sequence alignment was generated with the Clustal Omega program (http://www.ebi.ac.uk/Tools/msa/clustalo/) [24]. Protein structure prediction was performed with the Phyre2 online program (www.sbg.bio.ic.ac.uk/phyre2/) [25] and binding site predictions were carried out with 3DLigandSite (http://www.sbg.bio.ic.ac.uk/3dligandsite/) [26]. Other amino acid sequences of *E*. *granulosus*, homologous to Kunitz proteins, were searched by blast using the GeneDB online database (http://www.genedb.org/Homepage/Egranulosus). Cladogram phylogenetic analysis was performed with Phylogeny.fr (http://www.phylogeny.fr/) [27].

PCR primers with introduced N-terminal 6×His tag and NcoI and EcoRI restriction sites, (*EgKI-1*: FW- CATGCCATGGCACATCATCATCATCATCACGAAGAGGATGTCTGCAACCTACC, RV- GATCGAATTCCTATAAGTGCAAATTTTTAACACAAGCAC; *EgKI-2*: FW- CATGCCATGGCACATCATCATCATCATCACCTTCACAGAGACTGCAAGGATCC, RV- GATCGAATTCCTAGGCGATGGAGCATTGG) were designed and produced by Sigma Aldrich (St. Louis, MO, USA). Both *EgKI-1* and *EgKI-2* were PCR-amplified using cDNA from adult worms and MyTaq DNA polymerase. Purified PCR products were digested with the restriction enzymes *Nco*I and *EcoR*I and ligated into the pET28a expression vector. Recombinant protein production was induced after transforming the plasmids containing the *EgKI* genes into *E*. *coli* BL21 (DE3) cells.

Recombinant protein production, refolding and purification were carried out as described [11]. The concentrations of purified EgKI-1 and EgKI-2 were determined using the Bradford assay and the proteins were stored at -80°C. Aliquots of the recombinant EgKI proteins were subjected to electrophoresis on 15% (w/v) sodium dodecyl sulphate (SDS) polyacrylamide gels and stained with Coomassie Blue to determine their purity and relative molecular mass.

### Real time PCR

Preparations of cDNA from adult worms (AW), protoscoleces (PSC), hydatid cyst membranes (HCM) and oncospheres (ONC) were used for real time PCR (qPCR); primers (*EgKI-1*: FW- CGAAGAGGATGTCTGCAACC, RV- TCCACAACCACCGTAGATGA; *EgKI-2*: FW- ACTGCAAGGATCCCATTGAC, RV- TCCTCCAGCGTCTCAAAGTT) were designed using the online primer design software, Primer3 (http://simgene.com/Primer3). Each cDNA sample (25 ng per reaction) was tested in quadruplicate and all reactions were performed twice. *E*. *granulosus* eukaryotic translation initiation factor (*Eg*-*eif*) was used as housekeeping gene for the normalization of data. The confidence threshold (CT) of the second results set was normalized to the first set before evaluation by importing the standard curve of the first set, to the second. The results were analyzed using Rotor-Gene 6000 software.

### Production of polyclonal antibodies against EgKI-1 and EgKI-2 proteins

Purified recombinant EgKI-1 and EgKI-2 were dialyzed in PBS using 3500 MWCO Slide-A-Lyzer dialysis cassettes following the manufacturer’s instructions. Antiserum production was undertaken using six Swiss mice per protein. For the first immunization each mouse was inoculated subcutaneously with 50 μg of protein (dissolved in 50 μl PBS) emulsified with an equal amount of Freund’s complete adjuvant. Subcutaneous boosts of 50 μg of protein (dissolved in 50 μl PBS), emulsified with the same amount of Freund’s incomplete adjuvant, were undertaken on two occasions at two weekly intervals. Mice were bled for serum one week after the third immunization and the presence of serum anti-EgKI antibodies was confirmed by Western blotting. EgKI-1, EgKI-2, BPTI (Roche diagnostics, Mannheim, Germany), urinary trypsin inhibitor (UTI) (Prospec-Tany TechnoGene Ltd, Ness Ziona, Israel) and SmKI protein, 0.5 μg each, were fractioned on a 15% (w/v) SDS-PAGE gel and transferred to Immun-Blot low fluorescence-PVDF membrane. Overnight blocking was performed with Odyssey buffer at 4°C. Then, the membrane was subjected to incubation with the mouse anti-EgKI-1 or -EgKI-2 anti-serum (1:2,000 dilution in Odyssey buffer and 0.1% Tween-20) for 1 h followed by incubation with IRDye-labeled rabbit anti-mouse antibody (LI-COR Biosciences) (1:15,000 diluted in Odyssey buffer with 0.1% Tween-20 and 0.01% SDS) for 1 h on a shaker in a dark chamber. After a final wash with distilled water, the membrane was allowed to dry and visualized using the Odyssey imaging system.

### Western blotting and immunolocalization

Western blotting was carried out with both anti-EgKI-1 and anti-EgKI-2 antibodies using soluble native extracts of AW, PSC, HCM and ONC as described [28]. Paraffin blocks were made by embedding AW, HCM and PSC fixed in 10% phosphate buffered formalin in wax-filled moulds. Sections (4 μm) of these paraffin blocks were then adhered onto microscope slides. Following de-paraffinization and rehydration, antigen retrieval was done with RevealtA solution (Biocare Medical, Concord, CA, USA). Then the tissue sections were blocked with 1% (v/v) bovine serum albumin in Tris buffered saline (TBS) for 1 h and incubated with the mouse anti-EgKI-1 or -EgKI-2 anti-serum (1:200) at 4°C overnight. After washing with TBS-T (TBS with 0.1% Tween20), the sections were incubated with Alexa Fluor 488 goat anti-mouse IgG (1:500) (Invitrogen, Carlsbad, USA) at 37°C for 60 min. Nuclei in the tissue sections were counterstained with DAPIgold (Invitrogen, Carlsbad, USA) and observed under an EVOS fluorescence microscope.

### Serine protease inhibition assays

The inhibitory activity of the two recombinant EgKI proteins was tested using several commercially available mammalian serine proteases. Enzyme and EgKI protein mixture (ranging from 20 pM to 200 mM) were first incubated together in 96 well plates at 37°C for 10 min. Subsequently a chromogenic or fluorogenic substrate was added at concentrations ranging from 100 mM to 5 μM and product release was measured using a plate reader every min for 30 min.

Bovine pancreatic trypsin, bovine pancreatic α-chymotrypsin and the fluorogenic substrates *N*_α_-Benzoyl-L-arginine-7-amido-4-methylcoumarin hydrochloride and N-Succinyl-Ala-Ala-Pro-Phe-7-amido-4-methylcoumarin were purchased from Sigma Aldrich (St Louis, USA). Trypsin and chymotrypsin assays were performed in 200 mM Tris-HCl (pH 8.2) containing 20 mM CaCl_2_ and 0.1% PEG 8000. The kinetic rate of substrate hydrolysis was measured at excitation/emission wavelengths of 370/460 nm with a fluorescence microplate reader. Inhibitory activity of porcine pancreatic elastase (PPE) was observed using the *Enzcheck* elastase assay kit (Life technologies, Carlsbad, USA) following the manufacturer’s instructions. Fluorescence signals were measured at 505/515 nm.

Neutrophil elastase, Cathepsin G and Proteinase 3, with corresponding substrates N-Methoxysuccinyl-Ala-Ala-Pro-Val-7-amino-4-methylcoumarin, Suc-Ala-Ala-Pro-Phe-pNA and Boc-Ala-Ala-Nva-SBzl, respectively, were purchased from Enzolifesciences (NY, USA). The neutrophil elastase (NE) inhibition assay was carried out with buffer containing 100 mM HEPES, 300 mM NaCl and 0.05% Tween-20 (pH 8) with 2.5 nM enzyme and fluorescence signals were detected at 370/460 nm. Cathepsin G activity was determined in 100 mM Tris-HCl, 1.6 M NaCl buffer (pH 7.5) with 100 nM enzyme and release of Pro-Phe-pNA was measured at 405 nm. Buffer containing 100 mM HEPES pH 7.5, 500 mM NaCl, 10% DMSO was used to detect Proteinase 3 activity and substrate hydrolysis was detected at 412 nm following the addition of 170 μM 5,5'-dithiobis (2-nitrobenzoic acid) (DTNB).

The percentage of the relative activity of the EgKI proteins was calculated using the formula:

Percentage of relative activity = (ΔRU of inhibitor/ ΔRU of enzyme control) × 100%

Δ Relative fluorescence/ absorbance unit (RU) = R_2_—R_1_, Readings R_1_ and R_2_ were taken at t_1_ and t_2_ time points respectively, when the reaction was in the linear range.

The data obtained with different substrate concentrations ([S]) and several inhibitor concentrations ([I], each EgKI protein) were fitted into the substrate-velocity curves of competitive enzyme inhibition. K_i_ values were calculated using Graph Pad Prism version 6.02 software by nonlinear regression according to the formula; v = V_max_ [S]/ K_m_ (1+ [I]/K_i_) + [S], where v is velocity, V_max_ is the maximum velocity in the absence of inhibitor, and Km is the Michaelis constant of the substrate. Values were corrected after subtracting background signals and all experiments were performed in triplicates.

### Coagulation assays

The clotting of blood involves multiple serine proteases so we performed two standard tests (activated partial thromboplastin time, APTT; and the prothrombin time, PT) to determine whether the two recombinant EgKIs had any effect on the intrinsic and the extrinsic pathways of coagulation. Fresh healthy human blood (30 ml) was collected into sodium citrate vacutainers and the plasma was separated. Then, 800 μl plasma was mixed with 50 μl of each EgKI protein (final concentrations of 200 pM, 200 nM and 2 μM for EgKI-2 and 200 pM, 200 nM and 10 μM for EgKI-1) and incubated in a 37°C water bath for 10 min. After adding CaCl_2_ to the mixture, the time taken for clot formation was measured by a Sta-R coagulometer (Diagnostica stago, Asnières, France). TriniCLOT APTT HS (Trinity Biotech, Bray, Co Wicklow, Ireland) and Thromborel S (Siemens, Malvern, PA, USA) kits were used for the determination of the APTT and the PT, respectively. Aprotinin (Sigma Aldrich, St Louis, USA) and FVII negative plasma (Helena Laboratories, Texas, USA) were used as positive controls for APTT and PT, respectively.

### Calcium binding assay

As the 3DLigandsite predicted that EgKI-1 would bind calcium, a calcium binding assay was carried out using a published procedure [29] with minor modifications to confirm the prediction. Recombinant EgKI-1, recombinant EgKI-2 and bovine serum albumin (BSA) (New England BioLabs), as positive control, were separated on 15% (w/v) SDS-PAGE gels and the electrophoresed proteins transferred to a PVDF membrane. The membrane was washed with wash buffer (10 mM imidazole, 60 mM potassium chloride, 5 mM magnesium chloride, pH 6.8) for 1 h at 37°C with gentle shaking. After rinsing with distilled water, the blot was incubated with 1 mM CaCl_2_ for 1 h. Following washing (3X) with 20% (v/v) ethanol and a final wash with distilled water, the membrane was incubated with 1 mM Quin-2 (AM) (Sigma Aldrich) for 1 h. Subsequently the membrane was washed with distilled water and visualized using a UV transilluminator (Vilber Lourmat) at 365 nm. Quin-2 emits fluorescence signals in the presence of Ca^++^, enabling detection of proteins on the PVDF membrane bound to calcium.

### Carrageenan mouse air pouch model of local inflammation

The subcutaneous air pouch model, which is an *in vivo* model that can be used to study acute and chronic inflammation, was used according to a published protocol [30]. Briefly, female BALB/c mice weighing <25g were anesthetized with isoflurane and a subcutaneous dorsal pouch was made in each animal by injecting 3 ml sterile air. The pouch was reinjected with 1.5 ml sterile air, after two days. On the sixth day, a 15 μM protein sample (rEgKI-1 or the anti-inflammatory drug Ulinastatin [Prospec-Tany TechnoGene Ltd, Ness Ziona, Israel] as positive control: 200 μl) in PBS or PBS alone as negative control was injected into the pouch. An LPS control (L2880; Sigma Aldrich) was also included and consisted of 12 μg of LPS, which was an amount equivalent to the calculated LPS contamination of the bacterially produced rEgKI-1 protein measured using a Pierce LAL Chromogenic Endotoxin Quantitation Kit (Thermo Fisher Scientific Inc., IL, USA) with a sensitivity of 0.1 EU/ml (approximately 0.01 ng endotoxin per ml). After 30 mins, 300 μl of 1% (w/v) lambda (λ) carrageenan (Sigma Aldrich), which acts as an inflammatory stimulus [31], in sterile saline, was injected into the air pouch. On the following day, mice were euthanized and the pouches were washed with 1 ml ice cold lavage solution (0.5% EDTA in 0.9% saline). The collected lavage solution was immediately placed on ice and then centrifuged at 200 g for 10 min at 4°C. The resulting cell pellet was then resuspended in 500 μl lavage solution. Thin smears were made on microscope slides with the cell solution, stained with Diffquick stain and examined under x100 magnification. Differential cell counts were performed with the stained thin smears by counting 300 cells in total with the percentage of neutrophils in each sample calculated. The total cell count in the lavage solution was determined using a hemocytometer. Then, the total neutrophil count per mouse in the lavage solution was calculated by multiplying the percentage of neutrophils by the total cell count. The experiment was performed twice and in each case eight mice per group were used. Statistical analysis was performed with one-way analysis of variance (ANOVA) using GraphPad prism 6. Statistical significance was established at P < 0.05 compared with the PBS control and the LPS control.

### Ethics statement

Human venous blood was prepared from a healthy volunteer after he/she provided informed written consent and following review by the QIMRB Medical Research Institute (QIMRB) Human Ethics Committee. All animal experimentation was conducted in strict accordance with protocols approved by the QIMRB Animal Ethics Committee (project number P384), which adheres to the Australian code of practice for the care and use of animals for scientific purposes, as well as the Queensland Animal Care and Protection Act 2001; Queensland Animal Care and Protection Regulation 2002.

## Results

### Identification and analysis of Kunitz protein genes

**Fig 1:**
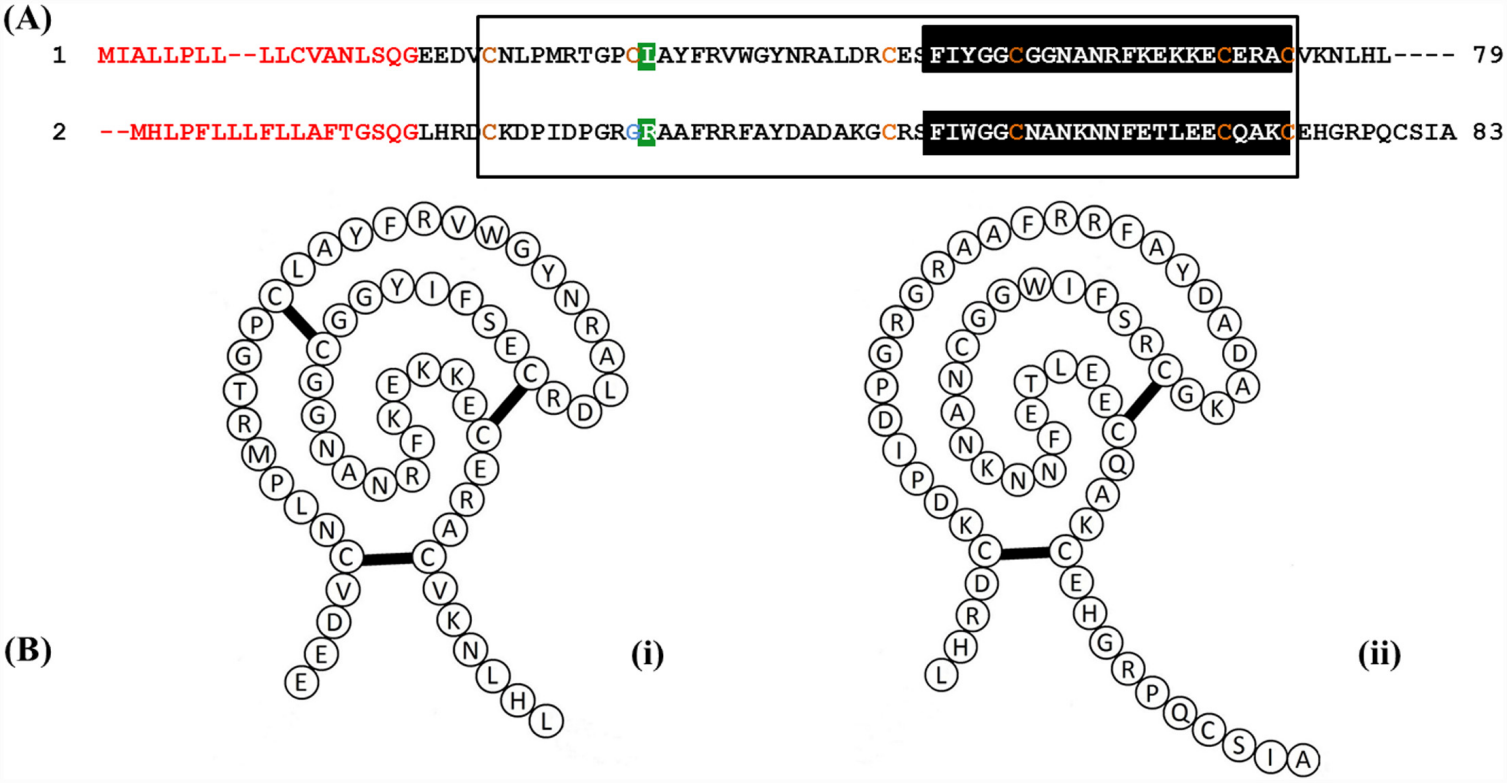
Amino acid sequences of EgKI-1 (1) and EgKI-2 (2). **A**. Signal sequences (18 amino acids) are in red, the Kunitz domain of both proteins is boxed and the Kunitz family signature highlighted in black. The conserved cysteine residues are shown in orange; EgKI-1 has six whereas EgKI-2 has five with one position replaced by a glycine (blue). The P1 reactive sites of both proteins are highlighted in green. B. Schematic diagram of (i) EgKI-1 showing three disulphide bridges, and (ii) EgKI-2 presenting two disulphide bridges.

**Fig 2:**
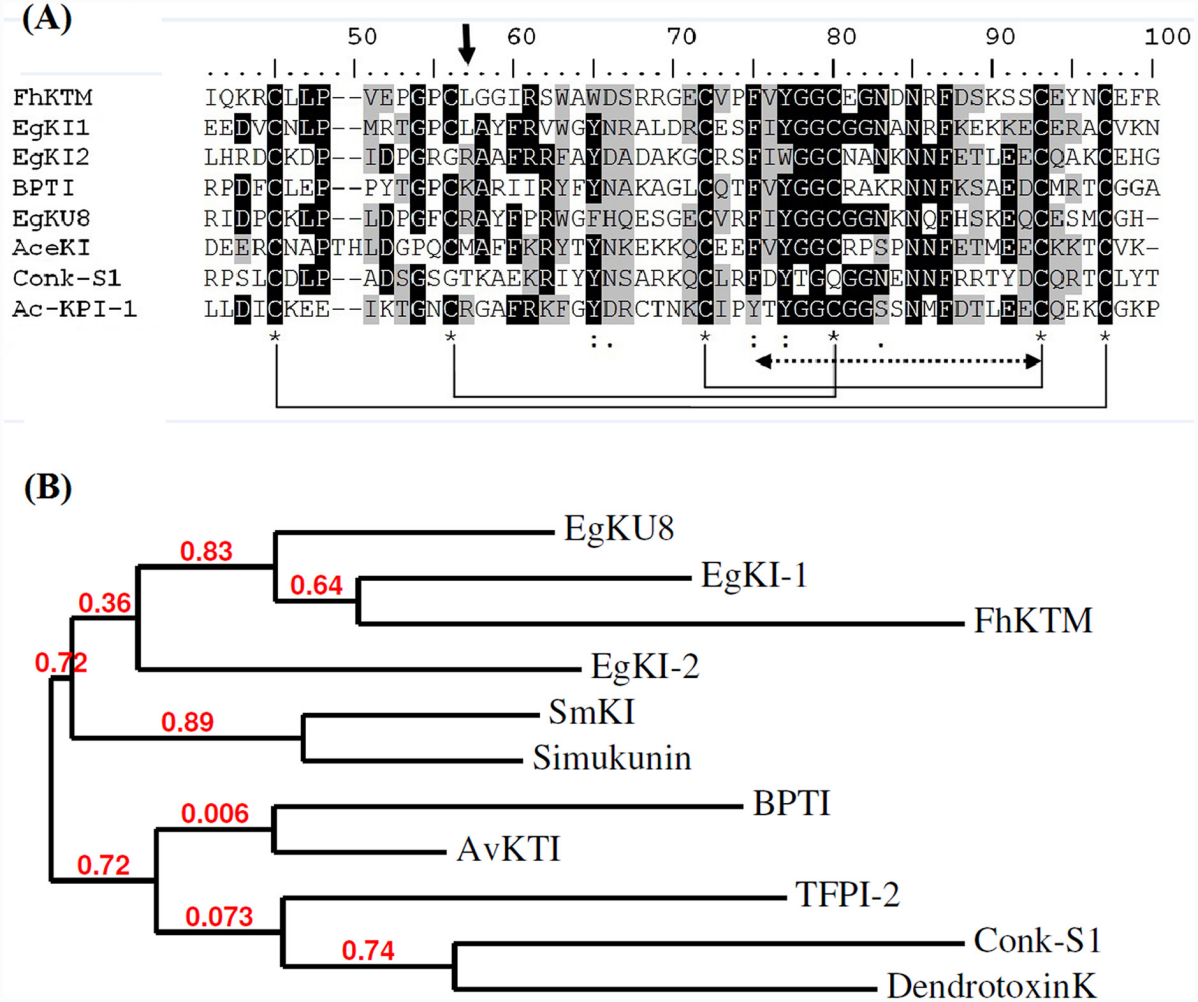
A. Partial amino acid sequence comparison of EgKI-1 and EgKI-2 with other Kunitz type protease inhibitors from: *Fasciola hepatica* (FhKTM, AAB46830.1); BPTI (1510193A); *Echinococcus granulosus* (EgKU8, ACM79010.1); *Ancylostoma ceylanicum* (AceKI, AAD51334.1); *Conus striatus* (Conk-S1, P0C1X2.1); the first domain of *Ancylostoma caninum* Kunitz inhibitor (Ac-KPI-1, AAN10061.1). The six conserved cysteine residues are marked by * and the pattern of disulphide bond formation is shown in brackets. The P1 reactive site is marked by the arrow head and the Kunitz family signature by the dashed double head arrow. B. Phylogenetic analysis of EgKI-1 and EgKI-2 with other Kunitz type protease inhibitors: FhKTM, EgKU8, BPTI, SmKI, Conk-S1, human tissue factor pathway inhibitor-2 (TFPI-2, AAA20094), Black fly (*Simulium vittatum*) (Simukunin, ACH56928.1), Orb weaver spider (*Araneus ventricosus*) (AvKTI, AFX95921.1), Black mamba (*Dendroaspis polylepis*) (DendrotoxinK, 1097974).

EG_08721 (GenBank: EUB56407.1), reported as being highly expressed in oncospheres and EG_07242 (GenBank: EUB57880.1), reportedly highly expressed in adult worms [4] were selected for further characterization and named *EgKI-1* and *EgKI-2* respectively. The full-length *EgKI*-1 and *EgKI-2* cDNAs have open reading frames of 240 and 252 nucleotides. Both translated peptides contain 18 amino acid signal sequences (Fig 1A) and have molecular weights of 8.08 kDa (EgKI-1) and 8.3 kDa (EgKI-2). EgKI-1 has six conserved cysteine residues whereas EgKI-2 has only five, apparently lacking the second disulphide bond (Fig 1B). A Clustal alignment comparison of EgKI-1 and EgKI-2 with well characterized Kunitz inhibitors available in GenBank revealed that the Kunitz family signature is highly conserved among different species (Fig 2A). Phylogenetic analysis of the two EgKIs (Fig 2B) confirmed their relatedness with Kunitz protein sequences from other taxa. Further interrogation indicated the presence of several other putative Kunitz inhibitors in the *E*. *granulosus* genome and transcriptome [4], suggesting these proteins likely play an important role in the parasite’s biology. Some of these Kunitz proteins contain a non inhibitory P_1_ amino acid whereas others contain a protease inhibitory amino acid. Also, several contain only Kunitz domains, whereas some associate with other domains such as Ig or spondin. Clustal alignment performed with the ten putative single domain Kunitz inhibitors of *E*. *granulosus* identified nine typical trypsin inhibitors and one typical chymotrypsin inhibitor (S1 Fig).

### Real time PCR

**Fig 3:**
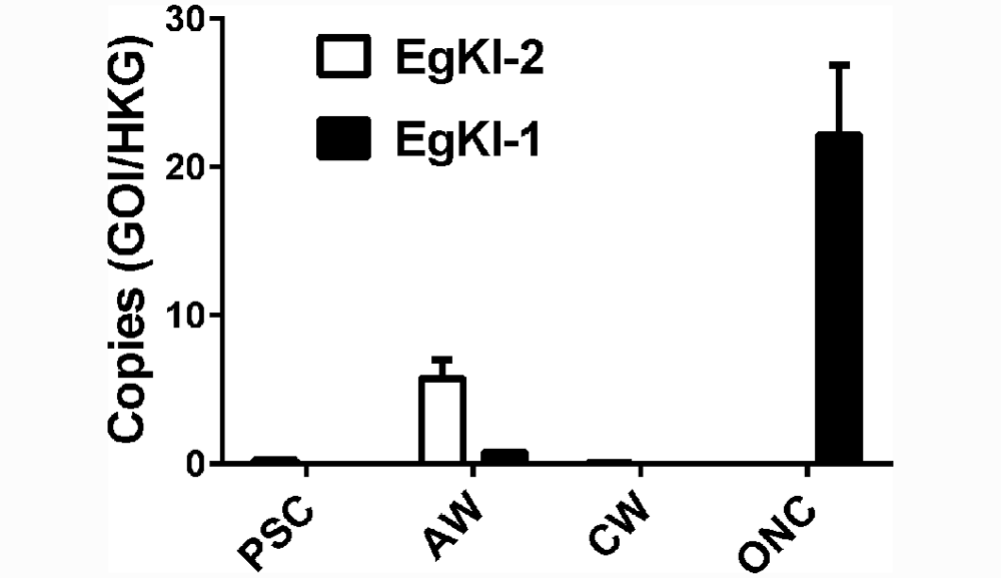
Normalized expression levels of the EgKI genes in *E*. *granulosus*. The Y axis represents the number of copies after dividing the number of copies of either gene of interest (GOI) by the number of copies of the house keeping gene (HKG). PSC, protoscoleces; AW, adult worms; HCM, hydatid cyst membrane; ONC, oncospheres. Error bars represent the mean ± SEM.

*EgKI-1* was highly expressed in oncospheres, the infective stage of *E*. *granulosus* for humans and intermediate hosts, such as sheep, whereas *EgKI-2* was more highly expressed in adult worms compared with *EgKI-1* (Fig 3).

### Protein expression, western blotting and immunolocalization

**Fig 4:**
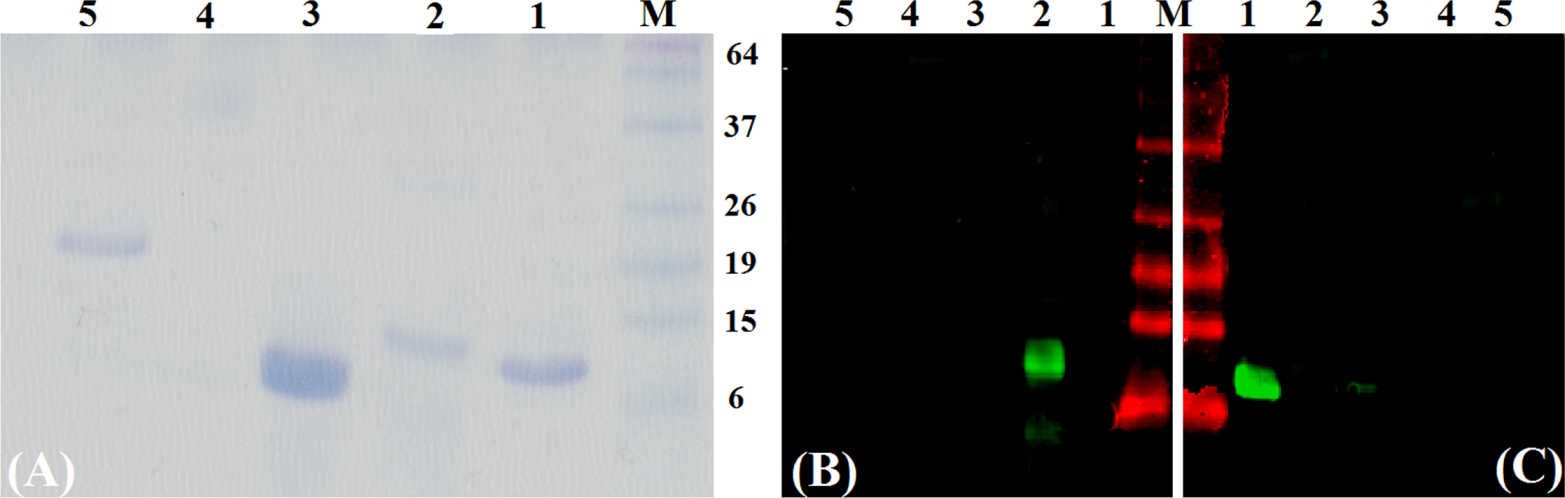
**(A) SDS-PAGE analysis and Western blotting with mouse antisera raised against (B) EgKI-2 and (C) EgKI-1 proteins: Lane 1, EgKI-1; Lane 2, EgKI-2; Lane 3, BPTI; Lane 4, UTI; Lane 5, SmKI; Lane M, protein marker**. Aliquots of 3 μg from each protein sample were used for SDS-PAGE and 0.5 μg for western blotting.

**Fig 5:**
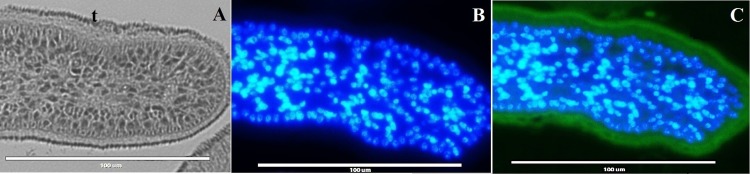
Immunolocalization of EgKI-2 in histological sections of an adult worm of *E*. *granulosus* (A) Bright field, (B) probed with pre-immune sera as negative control, (C) probed with EgKI-2 immunized mouse serum (1:200). DAPI counterstained nuclei are stained blue and the positive green fluorescence marks the presence of EgKI-2 along the tegument (t). Scale Bar = 100 μm.

Purified yields of 1 mg/ L and 0.4 mg/ L, respectively, were obtained for recombinant EgKI-1 and EgKI-2 (Fig 4A). There was no cross immuno-reactivity between EgKI-1 and EgKI-2 as shown by western blotting of the recombinant proteins with antisera from mice immunized with the two proteins; moreover, neither antisera reacted with *E*. *coli* produced SmKI or to the mammalian Kunitz proteins, BPTI and UTI (Fig 4B and 4C). There was no positive reactivity with soluble antigen extracts from AW, PSC, HCM or ONC by either the anti-EgKI-1 or anti-EgKI-2 murine antisera in western blots. A positive reaction was evident with the EgKI-2 anti serum along the tegument of sections of adult worms (Fig 5) but no positive reactivity was observed with sections from any of the life cycle stages probed with the EgKI-1 antiserum.

### Calcium binding assay

**Fig 6:**
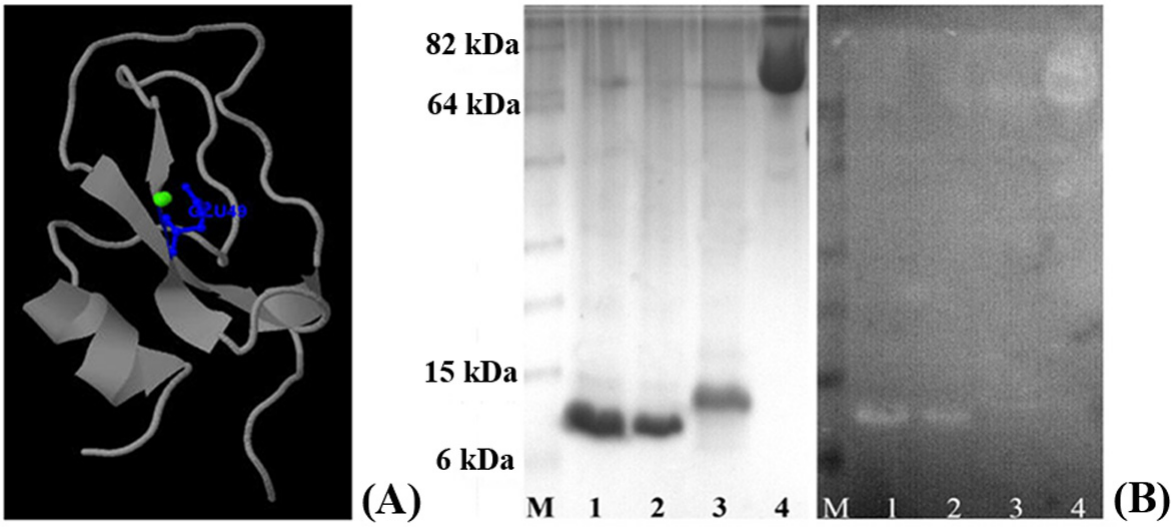
**(A) Predicted calcium binding site of EgKI-1, shown in blue**. (B) Calcium binding assay: SDS-PAGE gel (left) and corresponding western blot membrane of the calcium binding assay showing clear bands corresponding to the EgKI-1 protein: M-Marker; 1, 6 μg EgKI-1; 2, 2 μg EgKI-1; 3, 6 μg EgKI-2; 4, 6 μg BSA.

According to the 3DLigandsite prediction, calcium ions bind with the glutamine (Glu) residue at position 49 in EgKI-1 (Fig 6A). Recombinant EgKI-1 is shown to bind calcium more strongly than EgKI-2 (Fig 6B).

### Protease inhibition and coagulation assays

**Fig 7:**
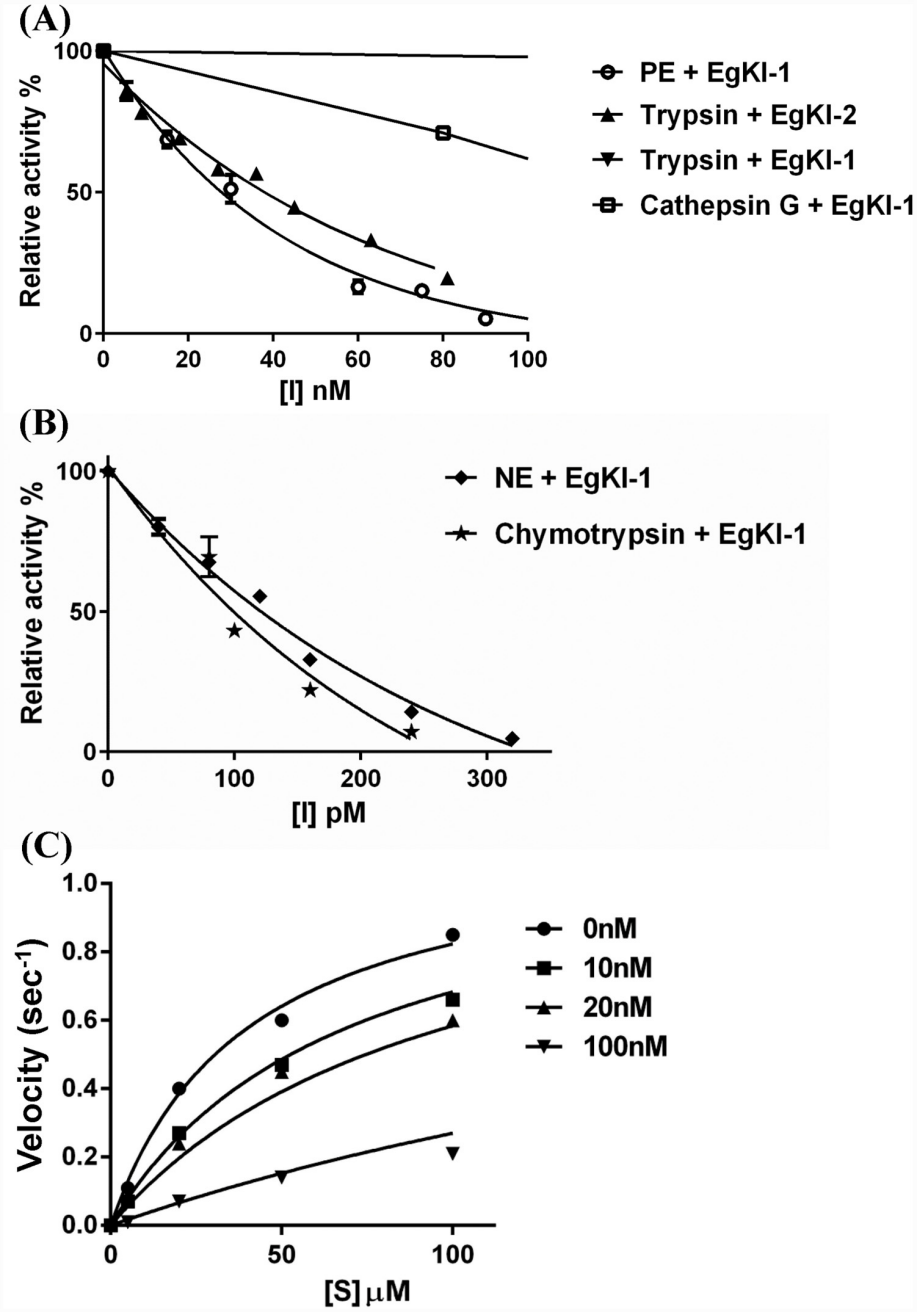
Inhibition of different serine proteases with increasing concentrations of the EgKI proteins. The relative activity (as a %) with: **(A)** nanomolar (nM) concentration range of EgKI-1 with trypsin, pancreatic elastase and cathepsin G, and EgKI-2 with trypsin **(B)** picomolar (pM) concentration range of EgKI-1 with neutrophil elastase and chymotrypsin. **(C)** Progress curves for trypsin inhibition with increasing concentrations of EgKI-2 (0 nM, 10 nM, 20 nM, 100 nM) with different substrate concentrations ([S]).

In serine protease inhibition assays (Table 1 and Fig 7) EgKI-2 reacted as a typical trypsin inhibitor, having no significant inhibitory activity against the other tested proteases. EgKI-1 inhibited all tested proteases, except proteinase3, showing a high potency for inhibiting chymotrypsin and neutrophil elastase (Table 1 and Fig 7). Varying the pre-incubation time period of the EgKI proteins with the serine proteases did not affect their inhibitory capacity suggesting that they are not “slow binders” [32]. Moreover, the denatured EgKI-1 and EgKI-2 proteins in 4 and 8 M Urea showed no or only minor inhibitory activity indicating the necessity of correct folding for inhibitory function (S2 Fig).

**Table 1:** Inhibitor constant (K_i_) values in Molar (M) range of EgKI-1 and EgKI-2.

Inhibitor	Trypsin		Chymotrypsin		Pancreatic elastase		Neutrophil elastase		Cathepsin G	
	Ki (M)	CI_95_	Ki (M)	CI_95_	Ki (M)	CI_95_	Ki (M)	CI_95_	Ki (M)	CI_95_
EgKI-1	2.32x10^-7^	0.17–0.28	1.484x10^-9^	0.20–0.62	1.65x10^-9^	1.23–2.06	6.42x10^-11^	32.74–95.66	4.47x10^-10^	0.20–0.62
EgKI-2	1.38x10^-8^	9.84–17.8	NI	NI	NI	NI	NI	NI	NI	NI

NI- No inhibition,

CI_95_- 95% confidence interval.

Neither recombinant EgKI-1 nor EgKI-2 interfered with the blood coagulation pathway when tested for APTT and PT. Neither protein prolonged the time taken for clot formation in either test, indicating no or minimal inhibition of proteases involved in the coagulation cascade (S3 Fig).

### Mouse air pouch model

**Fig 8:**
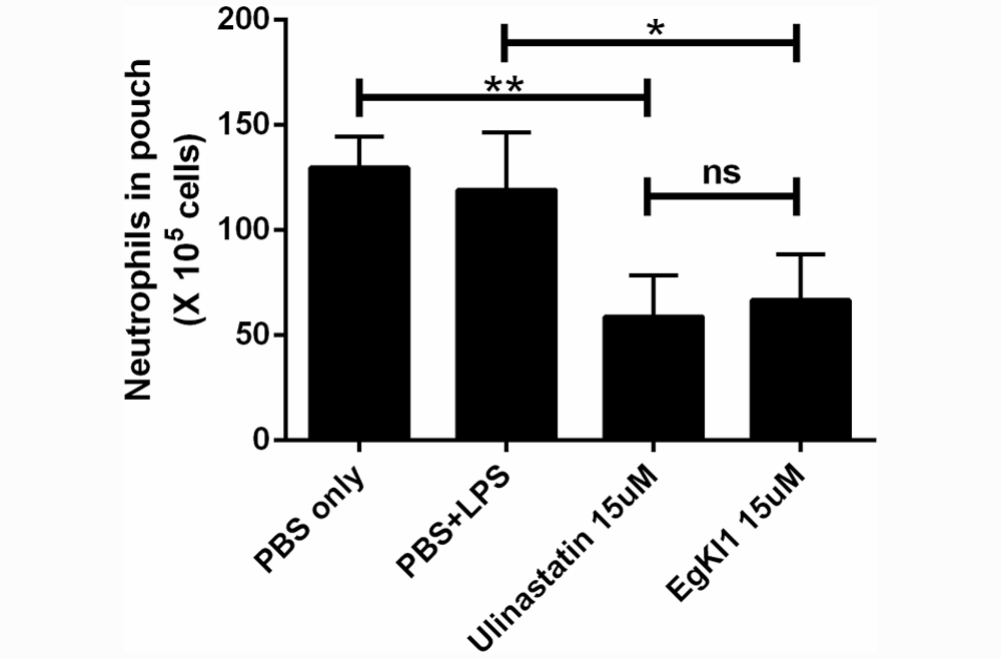
Inhibition of neutrophil chemotaxis by EgKI-1 in the mouse air pouch model. The data represent the mean ± SEM values of groups of eight mice in two independent experiments and analysis by one-way ANOVA. * P value, 0.03; ** P value, 0.001; ns, not significant (p value ≥ 0.05).

The results of the mouse air pouch model indicated that the infiltration of neutrophils to the inflammatory site was significantly reduced by around 50% (P value <0.05) in the presence of 15 μM EgKI-1or the positive control Ulinastatin compared with the PBS control; injection of the LPS control had no effect on the numbers of neutrophils infiltrating the pouch (Fig 8).

## Discussion

The host-parasite relationship is complex being mediated both by parasite virulence factors and exacerbated by host responses. The presence of Kunitz type proteins in numerous phylogenetically diverse species suggests that these molecules perform important biological functions even though their precise role in each organism is not yet fully understood. We focused on two nucleotide sequences (*EgKI-1* and *EgKI-2*) encoding secreted single domain Kunitz type protease inhibitors (EgKI-1 and EgKI-2) from *E*. *granulosus*, that we had identified by interrogation of the available genomic sequence. EgKI-1 has all six conserved cysteine residues present giving rise to three disulphide bonds (C1-C6, C2-C4 and C3-C5) but EgKI-2 lacks the 2^nd^ disulphide bond (C2-C4). Previous studies have shown that the lack of the C2-C4 bond and even selective inhibition of the same bond has no effect on the stability of Kunitz proteins or their inhibitory properties [33, 34]. Conkunitzin-S1 is a neurotoxin from the venom of the cone snail *Conus striatus* which, despite missing the C2-C4 bond, retains its functional activity [35]. Furthermore, similar to EgKI-2, Huwentoxin-XI (HWTX-XI) from the venom of tarantula spiders (*Ornithoctonus sp*), is a potent trypsin inhibitor despite lacking the 2^nd^ disulphide bond [36].

Real time PCR showed *EgKI-1* is highly expressed in the oncosphere which is the stage infective to humans and ungulate hosts. The fact that EgKI-1 inhibited the activities of trypsin, chymotrypsin and PPE may be a feature that helps protect oncospheres from digestion by these enzymes in the small intestine of susceptible mammals. In contrast *EgKI-2* is highly expressed in adult worms and, as shown by immunofluorescence, the protein is localized to the tegument, suggesting a possible role in protecting the parasite from the constant trypsin exposure that it is subjected to in the small intestine of the canine definitive host. Due to the considerable risk of handling and the difficulties in obtaining material, immunolocalization of the two EgKIs in activated oncospheres was not possible. The fact that neither murine anti-EgKI-1 nor anti-EgKI-2 antisera showed any positive reactivity with any of the western blot-tested soluble antigen extracts from different life cycle stages of *E*. *granulosus* suggests that either both EgKI proteins are produced in low quantity or are only expressed following an external stimulus, such as when the worm comes in contact with host proteases. A recent transcriptomic study identified five protease inhibitors, including EgKU8 [37], in the excretory/secretory products of *E*. *granulosus* protoscoleces but more sensitive technologies may be required to identify other proteins expressed at lower levels of abundance as may be the case with EgKI-1 and EgKI-2.

The two Kunitz proteins were recombinantly expressed and purified by column refolding from induced lysates of *E*. *coli* cells transformed with *EgKI*/pET28a plasmids. Refolding of bacterially produced protein inclusion bodies immobilized by nickel chelating chromatography is a proven method for reconstituting the native properties of recombinant proteins and making them suitable for structural and functional analysis [38]. As a result, we were able to show that both EgKI proteins are potent serine protease inhibitors. Nanomolar range inhibition of trypsin activity was disclosed for EgKI-2, while EgKI-1 inhibited chymotrypsin and neutrophil elastase in the picomolar range. The specificity of a protease inhibitor against a protease is mainly determined by the nature of the amino acid residue at position P_1_ of its active site. The results we obtained with the EgKI proteins are in agreement with previous findings of Kunitz inhibitors from other taxa, where typical trypsin inhibitors have Arg (R) or Lys (K) at P_1_, and chymotrypsin inhibitors have Leu (L) or Met (M) [39]; EgKI-1 and EgKI-2 have Leu and Arg residues at the P_1_ site, respectively. The EgKIs are likely to play an important role in *E*. *granulosus* survival within their mammalian hosts and thus have potential as new drug and/or vaccine targets as control interventions. EgKI-1 may prevent the oncosphere from being digested in the gut by inhibiting trypsin, chymotrypsin and pancreatic elastase before it penetrates the intestinal wall. Similarly, trypsin inhibition by EgKI-2 may help provide protection to the adult worms while residing in the small intestine of the canine host.

Inflammatory responses occur after surgery, trauma and infection, and involve neutrophil activation and infiltration into the injured tissue. Neutrophil infiltration also occurs in the early stages of echinococcal infection [40]. Activated neutrophils release proteases such as neutrophil elastase, cathepsin G and proteinase 3 which, if not appropriately controlled, can result in severe damage to healthy tissue. Uncontrolled proteolysis can lead to various diseases/disease syndromes including emphysema, idiopathic pulmonary fibrosis, respiratory distress syndrome, cystic fibrosis, rheumatoid arthritis and glomerulonephritis [41]. Neutrophil elastase is the major protease responsible for extracellular proteolysis and it plays a pivotal role in the inflammatory response [42]. By releasing neutrophil elastase in the presence of foreign material in blood, infiltrating neutrophils activate a signalling pathway which triggers macrophages to secrete cytokines as well as to attract more neutrophils [43].

The most potent, specific human neutrophil elastase inhibitor described to date is a protein engineered from the Kunitz domain of human inter α inhibitor (EPI-HNE-4), which was shown to have a K_i_ value of 5.45 x 10^−12^ M [44]. Excessive accumulation of neutrophil elastase in pulmonary fluids and tissues of patients with cystic fibrosis (CF) is thought to act on the lungs, compromising their structure and function, so that EPI-HNE-4 has been suggested as an anti-inflammatory compound for the treatment of CF [44]. We show here that EgKI-1 is also a highly potent inhibitor (K_i_ = 6.42x10^-11^ M) of neutrophil elastase and it thus warrants further investigation as a potentially effective therapeutic for treating acute and chronic inflammatory diseases.

Cathepsin G has chymotrypsin-like catalytic activity and also has potent pro-inflammatory activity [42]. Calcium mobilization is one of the earliest events that occurs with neutrophil activation and is a key factor for modulating numerous neutrophil biological responses [45]. Once stimulated, the intracellular Ca^++^ concentration within neutrophils rises rapidly due to mobilization of ions from intracellular pools and influxes from the extracellular medium [46]. As well as playing a role in preventing proteolytic damage to oncospheres by neutrophil elastase and cathepsin G once they enter the blood circulation, EgKI-1, being a secretory protein, may also act to suppress further neutrophil activation by binding calcium ions [45] in the extracellular medium, thus making them unavailable to neutrophils.

Neutrophil chemotaxis plays an important role in the inflammatory response and, when excessive or persistent, may augment tissue damage. The fact that neutrophils have a short life span of around 6–8 hours after purification from whole blood is a limitation for performing assays with primary neutrophils [47]. Resting neutrophils become primed and then mobilized to the site of infection which involves receptor activation and secretion of cytokines, chemokines and other components [41]. Because of this, the molecular properties of primed neutrophils are very different to their resting state. The regulatory functions of macrophages are also shared by primed neutrophils. Hence, *in vitro* experiments with freshly isolated neutrophils can often fail to recognize their full functional activity [41]. For a more complete understanding of the functional activities of EgKI-1, we undertook *in vivo* experiments using the carrageenan induced mouse air pouch model. Subcutaneous injections of air over several days cause morphological changes in the cellular lining of the pouch and resemble a synovial cavity [48]. As an irritant, λ-carrageenan induces localized inflammation characterized by the infiltration of cells and a marked increase in the production of biochemical mediators. Thus, this model has been proven for use in pre-clinical studies of anti-inflammatory drugs [30]. It is notable therefore that EgKI-1 significantly reduced neutrophil infiltration to the inflammatory site, most likely as a result of its inhibition of neutrophil elastase. However, further studies are needed for a more complete understanding of the activity of EgKI-1 against inflammatory cytokines.

There is much current interest in developing novel potent drugs as treatments for inflammatory-related diseases to inhibit excessive and uncontrolled neutrophil serine protease activity [49, 50]. Clinical therapies that utilize protease inhibitors in controlling sepsis are currently restricted to the use of urinary trypsin inhibitor (UTI), mainly in Japan [51]. UTI, also referred to as ulinastatin or bikunin, is a multivalent Kunitz type serine protease inhibitor found in human urine and produces several anti-inflammatory effects [52]. Proteases also play important roles beyond their involvement in inflammation. In different types of cancers, the secretion of various proteases correlates with the aggressiveness of the tumour. Kunitz type inhibitors have been shown to exhibit promising anti-cancer properties which may be used in their development as novel cancer therapies [53]. Bikunin [54], TFPI-2 [55] and SPINT2 [56] are Kunitz proteins with anti-cancer effects, and as potent protease inhibitors, the EgKI proteins may also exhibit similar properties which can be exploited in cancer therapy.

In summary, this study further broadens knowledge of *E*. *granulosus* biology and emphasises the potential importance of the EgKI Kunitz proteins in protecting the worm by inhibiting or inactivating host digestive enzymes in the gut. As such they represent novel intervention targets for the control of cystic echinococcosis. Being a small molecule, a potent neutrophil elastase inhibitor and an inhibitor of neutrophil chemotaxis, EgKI-1 warrants further study as a potential therapeutic agent against inflammatory diseases [57]. As well, the potential anti-cancer effects of the EgKIs and their possible interactions with cytokines should be investigated.

## Supporting Information

S1 Fig Clustal alignment of the EgKI proteins with other single domain putative Kunitz type protease inhibitors identified in the *E*. *granulosus* genome. The P_1_ reactive sites are highlighted in black. Typical trypsin inhibitors have an arginine (R) at the P_1_ site whereas typical chymotrypsin inhibitors have a leucine (L).
(TIF)

S2 Fig Trypsin inhibition assays with denatured proteins (A) EgKI-1 (B) EgKI-2. (TIF)

S3 Fig APTT and PT with the EgKI proteins. (TIF)
