# Supplementary figures and images for: Cloning and Characterization of Two Potent Kunitz Type Protease Inhibitors from Echinococcus granulosus
Source: PLoS Negl Trop Dis. 2015 Dec 8;9(12):e0004268. doi: 10.1371/journal.pntd.0004268 (PMC4672886; doi:10.1371/journal.pntd.0004268)

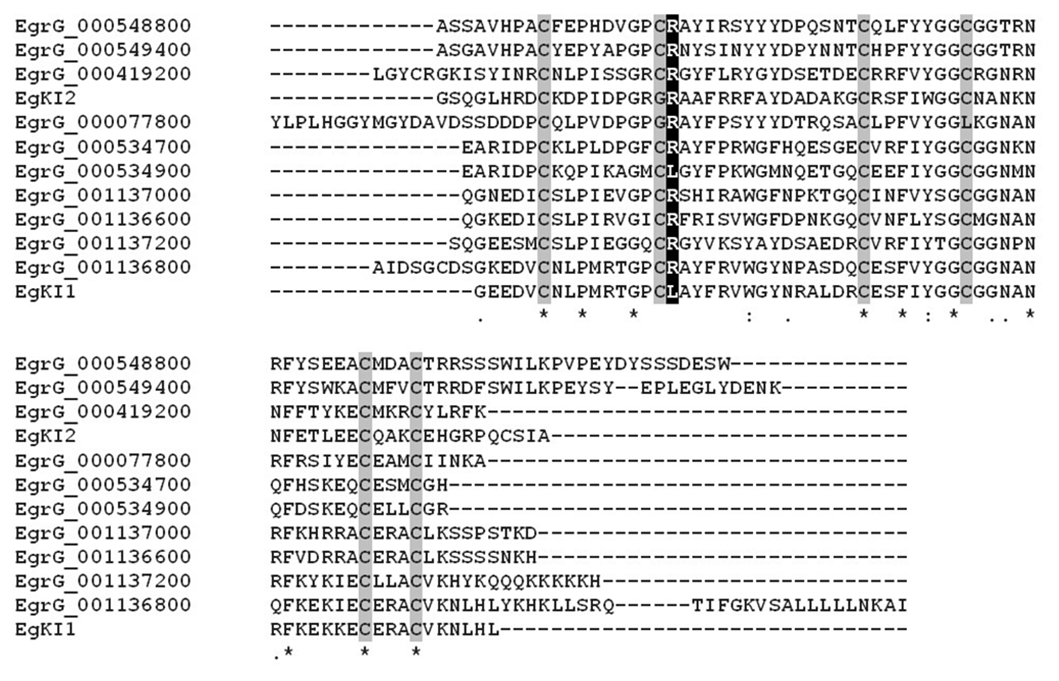

Supplement: S1 Fig — The P1 reactive sites are highlighted in black. Typical trypsin inhibitors have an arginine (R) at the P1 site whereas typical chymotrypsin inhibitors have a leucine (L). (TIF) [file pntd.0004268.s001.tif]

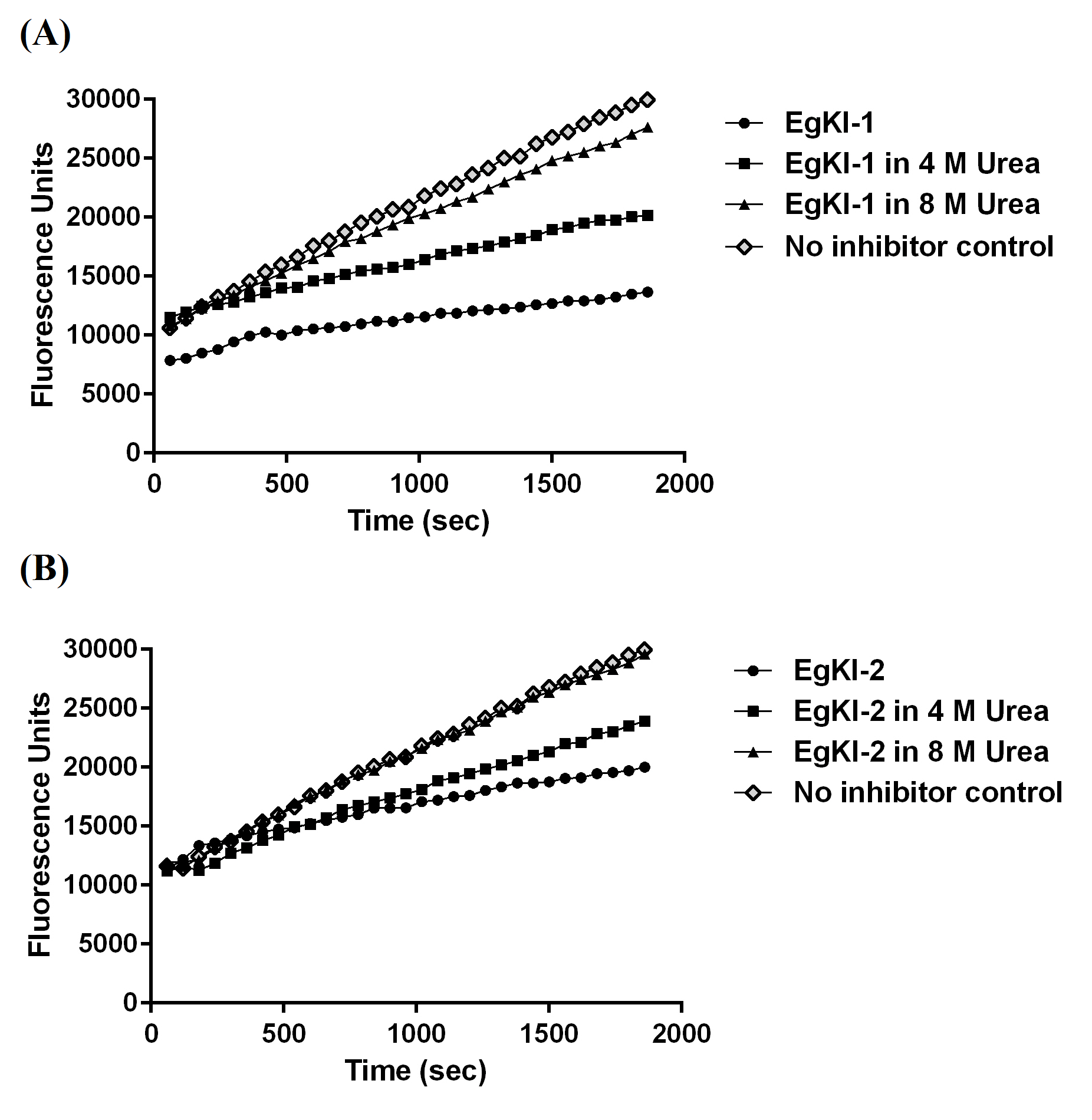

Supplement: S2 Fig — (TIF) [file pntd.0004268.s002.tif]

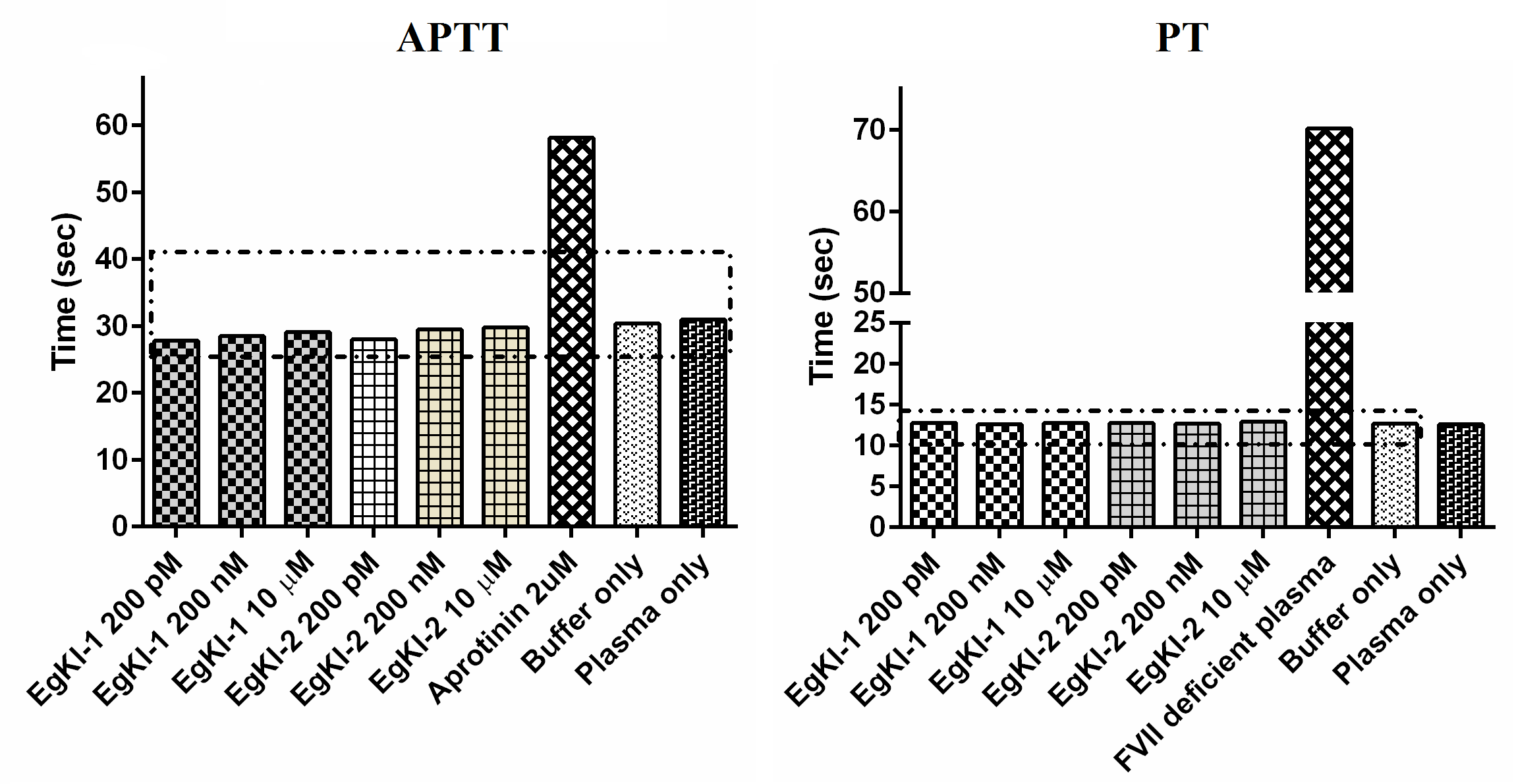

Supplement: S3 Fig — (TIF) [file pntd.0004268.s003.tif]
